# Deciphering the transcriptional circuitry of microRNA genes expressed during human monocytic differentiation

**DOI:** 10.1186/1471-2164-10-595

**Published:** 2009-12-10

**Authors:** Sebastian Schmeier, Cameron R MacPherson, Magbubah Essack, Mandeep Kaur, Ulf Schaefer, Harukazu Suzuki, Yoshihide Hayashizaki, Vladimir B Bajic

**Affiliations:** 1 South African National Bioinformatics Institute, University of the Western Cape, Modderdam Road, Bellville, South Africa; 2 RIKEN Omics Science Center, RIKEN Yokohama Institute, 1-7-22 Suehiro-cho, Tsurumi-ku Yokohama, Kanagawa, 230-0045 Japan; 3 Computational Bioscience Research Center (CBRC), King Abdullah University of Science and Technology (KAUST), Thuwal 23955-6900, Kingdom of Saudi Arabia

## Abstract

**Background:**

Macrophages are immune cells involved in various biological processes including host defence, homeostasis, differentiation, and organogenesis. Disruption of macrophage biology has been linked to increased pathogen infection, inflammation and malignant diseases. Differential gene expression observed in monocytic differentiation is primarily regulated by interacting transcription factors (TFs). Current research suggests that microRNAs (miRNAs) degrade and repress translation of mRNA, but also may target genes involved in differentiation. We focus on getting insights into the transcriptional circuitry regulating miRNA genes expressed during monocytic differentiation.

**Results:**

We computationally analysed the transcriptional circuitry of miRNA genes during monocytic differentiation using *in vitro *time-course expression data for TFs and miRNAs. A set of TF→miRNA associations was derived from predicted TF binding sites in promoter regions of miRNA genes. Time-lagged expression correlation analysis was utilised to evaluate the TF→miRNA associations. Our analysis identified 12 TFs that potentially play a central role in regulating miRNAs throughout the differentiation process. Six of these 12 TFs (ATF2, E2F3, HOXA4, NFE2L1, SP3, and YY1) have not previously been described to be important for monocytic differentiation. The remaining six TFs are CEBPB, CREB1, ELK1, NFE2L2, RUNX1, and USF2. For several miRNAs (miR-21, miR-155, miR-424, and miR-17-92), we show how their inferred transcriptional regulation impacts monocytic differentiation.

**Conclusions:**

The study demonstrates that miRNAs and their transcriptional regulatory control are integral molecular mechanisms during differentiation. Furthermore, it is the first study to decipher on a large-scale, how miRNAs are controlled by TFs during human monocytic differentiation. Subsequently, we have identified 12 candidate key controllers of miRNAs during this differentiation process.

## Background

The mononuclear phagocyte system is defined as a family of cells comprising of bone marrow progenitors and is derived from hematopoietic stem cells. Hematopoietic stem cells sequentially differentiate into monoblasts, promonocytes, monocytes and terminal macrophage cells [1]. The human monocytic leukemic cell line, THP-1 [2], is an accepted model system utilised to explore molecular events surrounding monocytic differentiation. Phorbol 12-myristate 13-acetate (PMA) induces the differentiation of monocytic THP-1 cells into macrophages/mature THP-1 cells [3]. Before inducing differentiation, PMA first inhibits cell growth and blocks THP-1 cells in G1-phase of the cell cycle by up-regulating the expression of p21^WAF1/CIP1^, enhancing binding of the SP1 factor to the p21^WAF1/CIP1 ^promoter. PMA inhibition of cell growth is mediated by several signalling pathways such as MAPK and ROS-dependent Raf/MEK/ERK pathway [4]. Human monocytic maturation incorporates lipid and protein metabolic processes together with several G-protein coupled receptors (GPCRs) [5].

Differential gene expression that results in human monocytic differentiation is regulated by numerous interacting transcription factors (TFs) [4-6]. Current research suggests that microRNAs (miRNAs) target several genes that are differentially expressed in the differentiation process [7]. miRNAs are ~22 nucleotides (nt) long non-coding RNAs, which play a key role in the repression of translation and degradation of coding mRNA [8-12]. Several computational tools are available for prediction of miRNA targets [9,13-16].

Canonical miRNA biogenesis begins with the transcription of the pri-miRNA by RNA polymerase II [17-19]. These pri-miRNAs are cleaved into 60~70 nt pre-miRNAs by the microprocessor complex Drosha (RNase II endonuclease) and DGCR8 (a double-stranded RNA binding protein) [20,21]. Pre-miRNAs are then exported to the cytoplasm with the help of Exportin-5 and its co-factor RanGTP [22]. Dicer, a RNase III endonuclease, cleaves 22-nucleotide from the Drosha cleavage site to yield the mature miRNA [8,23]. The generation of pri-miRNA by RNA polymerase II suggests that miRNA genes are controlled through the same regulatory machinery as the protein coding genes.

A straightforward analysis of the transcriptional regulation of miRNA genes is difficult. Even though most miRNAs have their own transcriptional units [8], it is known that several miRNAs are transcribed together as a single pri-miRNA [24-26]. These clustered miRNAs are thus co-regulated. On the other hand, miRNAs can also be transcribed together with a protein-coding host gene [8]. In addition, a mature miRNA can be produced from several locations in the genome [8,27]. Furthermore, it is not clear how to define the regulatory regions for miRNA genes. Current research suggests that at transcription start sites (TSSs) of genes, histones are generally trimethylated at lysine 4 residues [28,29]. This has led to a potential definition of promoter regions for miRNAs [30] in human embryonic stem cells using such determined TSSs as the reference points.

As the transcriptional regulation of miRNAs is not well understood, we focus our study on the analysis of transcriptional regulation of miRNAs during monocytic differentiation. Gene expression of miRNAs and TFs was measured prior to PMA stimulation and over a 96 hour time-course post-PMA stimulation. We first utilised a general method to identify miRNAs whose expression levels differed due to PMA stimulation in THP-1 cells. We extracted promoter regions for these miRNAs and computationally mapped TF binding sites (TFBSs) to the promoter sequences. We made use of a time-lagged expression correlation analysis [31,32] to evaluate the predicted TF→miRNA associations by combining our *in silico *TFBS analysis with the measured *in vitro *expression data. This kind of a time-lagged expression correlation analysis has been utilised before to either predict or score TF→gene or gene→gene associations [33-35]. From these TF→miRNA associations we identified 12 TFs likely to play a central role in regulating miRNAs throughout the considered differentiation process. Six of these 12 TFs (ATF2, E2F3, HOXA4, NFE2L1, SP3, and YY1) have not been previously described as important for monocytic differentiation. The remaining TFs, CEBPB, CREB1, ELK1, NFE2L2, RUNX1, and USF2, although known to be involved in monocytic differentiation, were not known to play role in transcription regulation of miRNAs in this process. We concluded the analysis by highlighting several inferred regulatory networks that suggest interplay of TFs, miRNAs, and miRNA targets and that are likely to have an impact on the differentiation process.

To the best of our knowledge this research is the first large-scale study that attempts to decipher the transcriptional circuitry that regulates the expression of miRNAs during human monocytic differentiation and identifies potential new avenues for further research.

## Results and Discussion

**Figure 1:**
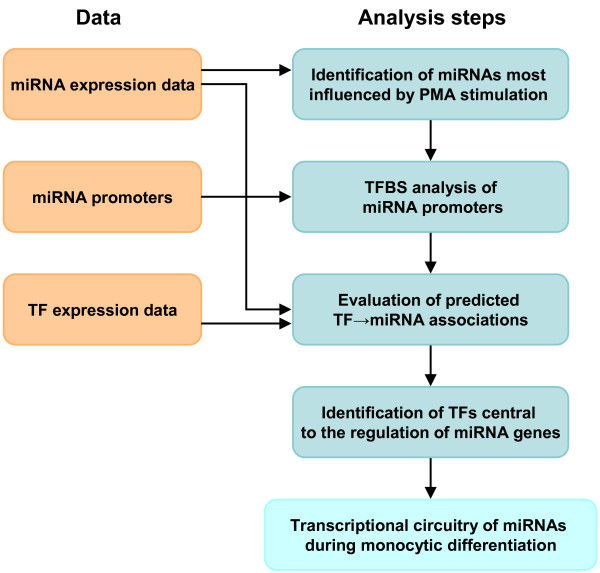
**Overview of the analysis**. The figure illustrates the analysis steps (blue/green boxes). In addition, the figure shows the data (red boxes) that has been utilised within individual analysis steps.

In what follows we present and discuss the main results of the study. Figure 1 gives an overview of the analysis steps. First, we analysed the miRNA expression data to identify miRNAs that are mostly affected by the PMA stimulation. We extracted promoter regions for the identified miRNAs and predicted TFBSs in these regions. Subsequently, we scored each predicted TF→miRNA association using a time-lagged expression correlation analysis to get a measure of reliability for the predicted associations. Afterwards, we statistically identified TFs that are likely to play a central role in regulating miRNAs during the monocytic differentiation process. Finally, for several miRNAs we investigated the predicted transcription regulations and their potential influence on the differentiation process.

### Identification of miRNAs most influenced by PMA stimulation

We are interested in the transcriptional regulation of those miRNAs whose expression is most influenced by the PMA stimulation. Three biological replicates of miRNA expression data provided measured expression levels at nine time-points after PMA stimuli and a zero hour control prior to PMA stimulation (see Methods). We required that two criteria were met for the inclusion of a miRNA expression time-series ('expression series' in further text) in the analysis:

i/ Expression of the miRNA should be denoted as "present" in at least one time point, otherwise we assume that the expression series for the miRNA is invalid. In this manner, we identified 155, 238, and 191 miRNAs and associated expression series for the first, second, and third replicate, respectively.

ii/ For a miRNA, i/ must hold true in at least two of the three biological replicates.

The expression values of different biological replicates for a miRNA that satisfy the criteria have been averaged at each time point to generate one expression series per miRNA. This resulted in expression series for 187 miRNAs (see Methods).

**Figure 2:**
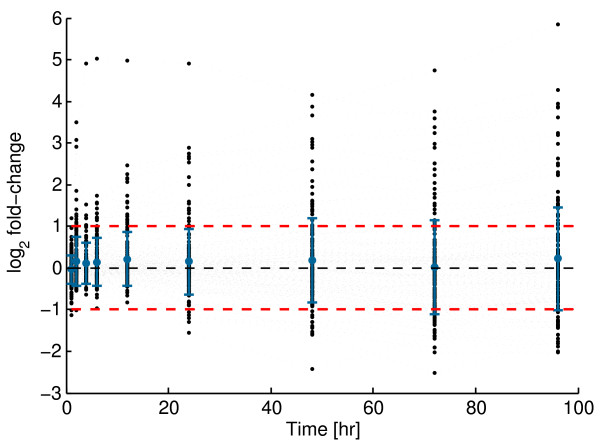
**Selecting PMA-induced miRNAs**. The figure illustrates for all measured time-points after PMA induction the log_2 _*fc *of the averaged expression set for all 187 selected mature miRNAs (black dots). Each dot represents a log_2 _*fc *of a single miRNA at the considered time point relative to the zero time point. The red dashed lines mark the log_2 _*fc *of 1 and -1 that are utilised as a cut-off for miRNAs (see main text). The figure shows in addition the mean (blue dot) and the standard deviation of all log_2 _*fc *values from the 187 miRNAs at the considered time point (blue error bars). Grey dashed lines indicate individual miRNA expression series. The figure shows that the majority of the miRNA expression does not change significantly over time and is confined within the selected threshold values.

In order to find the set of 'most relevant' miRNAs, we calculated for each of the 187 identified miRNAs the log_2 _*fc *(*fc *standing for fold-change relative to time zero) at each of the measured time points (see Methods). A miRNA we considered to be influenced by PMA stimulation if its log_2 _*fc *> 1 or log_2 _*fc *< -1 at any measured time point post-PMA stimulation (see Figure 2). Figure 2 shows that the majority of the miRNA expression does not change significantly over time and is confined within the selected threshold values. We found a total of 81 miRNAs that satisfied this criterion. To determine those miRNAs that deviate from the baseline expression we proceeded as follows. For each time point *t *where log_2 _*fc *> 1 or log_2 _*fc *< -1 was satisfied for a miRNA, we calculated the difference *d*_t _of the expression *e*_t _at time point *t *and its expression *e*_0 _at the zero time point. We sub-selected those miRNAs for which abs(*d*_t_) > 0.1 for at least one time point. This resulted in a set of 53 miRNAs for which we are more confident that their expression is affected by the PMA stimulation.

The *fc *does not take the level of expression into account. It is important to note that miRNAs that have very high expression level and change only little over time might have a strong biological impact, even though this is not reflected by variation in the expression level. Our approach, based on *fc *excludes such cases. On the other hand, miRNAs with very low expression levels might have high *fc *values that may suggest a strong biological impact, even though this may be arguable since the changes in expression levels could be very small. Hence, we introduced a second threshold for the difference in expression values of 0.1, even though no guideline exits for choosing this threshold.

### TFBS analysis of miRNA promoter regions

Promoter regions of miRNAs are regions of DNA where TFs bind to regulate the transcription of miRNA genes into pri-miRNAs. A pri-miRNA can be associated to several promoter regions derived from different TSSs. The transcriptional control of TFs is towards the pri-miRNA that can be cleaved into several pre-miRNAs [36]. Thus, we consider the miRNAs that form such clusters to be generally regulated in the same manner.

Marson *et al*. [30] defined promoter regions of miRNAs using TSSs determined based on trimethylated histones. We chose to analyze these promoter regions. For 34 of the 53 earlier identified mature miRNAs we were able to extract 38 promoter regions for 37 associated miRNAs (see Methods and Additional Files 1).

To map TFBSs to the 38 promoters we utilised TRANSFAC Professional database (version 11.4) [37,38]. TRANSFAC's 522 mammalian minimum false positive matrix profiles of binding sites were mapped to the promoter regions (see Methods). These matrices, which correspond to the predicted TFBSs, are associated with TFs that possibly bind these TFBSs (see Methods). By mapping the matrices to their corresponding TFs, we obtained 5,788 unique TF→miRNA associations for 673 TFs and 37 miRNAs.

### Evaluation of predicted TF→miRNA associations

Each predicted TF→miRNA association has been evaluated to get the most accurate picture of miRNA gene regulation during human monocytic differentiation. The result of this evaluation relates to our confidence that we are dealing with a genuine TF→miRNA association. The evaluation was based on time-lagged expression correlation between the gene expression series of the TF and that of the mature miRNA (see Methods). Expressions for miRNAs and TFs have been measured in human THP-1 cells prior PMA stimulus at one time point and post-PMA stimulus at non-equidistant time points up to 96 hours.

We interpolated the expression series for each of the 34 mature miRNAs using half an hour steps (see Additional Files 2). In concordance with the miRNA expression data, we averaged the TF qRT-PCR expression series over the two biological replicates at the same time points and interpolated each expression series using half an hour steps (see Methods). In this manner, we derived expression series for 2,197 TFs (see Methods).

**Figure 3:**
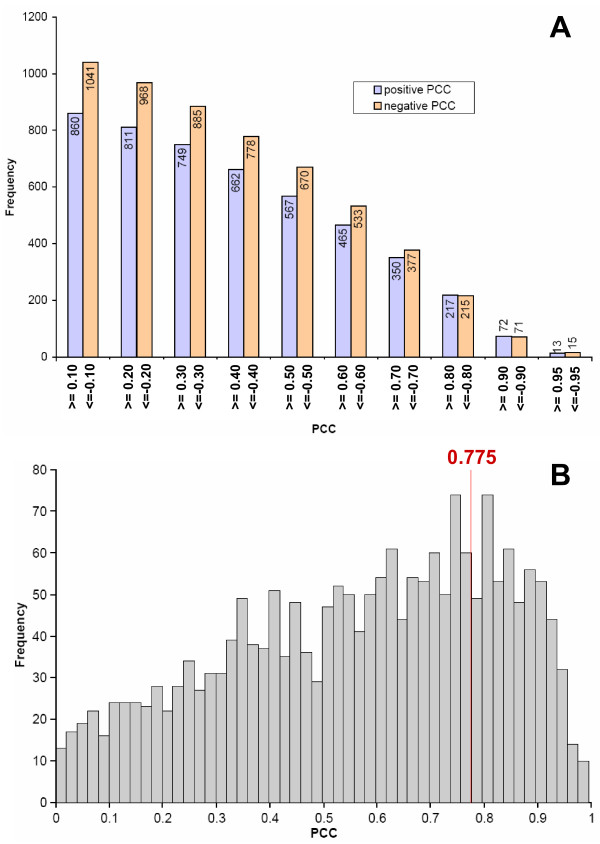
**TF→miRNA associations and their inferred Pearson correlation coefficients**. A/ Depicted is the number of TF→miRNA associations that have a score equal or greater then specific *PCC*s. The blue blocks indicate the number of associations that have a positive *PCC *greater or equal to the positive value indicated on the x-axis. The red blocks indicate the number of associations with a negative *PCC *smaller or equal to the negative value indicated on the x-axis. As expected, the number of associations steadily decreases with increasing absolute *PCC*. B/ Depicted is the distribution of the absolute value of the calculated *PCCs *for all 1,989 TF→miRNA associations. The red line indicates the cut-off value that was utilised to select the top quartile of the associations. The distribution is not normal distributed, but skewed towards higher *PCCs *resulting from the chosen method of time-shifts, which favours higher *PCCs *over lower ones.

The TF→miRNA associations were inferred from TFBS analysis of promoter regions of miRNA genes. From the predicted 5,788 TF→miRNA associations, we discarded all associations for which we do not have expression data for the TF in the above mentioned averaged expression set. After calculating Pearson's correlation coefficient (*PCC*) for each TF→miRNA associations using a time-lagged expression correlation analysis and the interpolated expression data for TFs and mature miRNAs, we finally derived a set of 1,989 TF→miRNA associations (see Additional Files 3) for 37 miRNAs and 258 TFs (see Additional Files 4), each associated with a *PCC *value (see Methods). In Figure 3A we show the number of TF→miRNA associations that have *PCCs *equal to or greater than selected thresholds. As expected, the number of associations steadily decreases with increasingly stringent *PCC *thresholds.

Previous research demonstrated that the regulatory effects of a TF on its target genes is not instantaneous but with a time-lag [39-41]. Unfortunately, the correct time-shifts are undetermined. In our analyses, we incorporated time-shifts in a range from 0.5 hours to six hours to allow for a sufficient time-delay for the regulation by the TF to exert an effect on the transcription of its target miRNA genes. We calculated for each of the 1,989 TF→miRNA associations the most favourable time-shift and with this, the time-lagged *PCC *of expression as the score for the association (see Methods). The higher the absolute value of the *PCC *for an association, the more confidence we have that the association is genuine and could play an important role in the differentiation process. For each miRNA/miRNA-cluster and its regulating TFs, the maximum *PCC*s were calculated individually (see Methods). Other approaches considered all TFs that regulate a gene to extract a common time-shift for all TFs and the gene [33] or compute the best time-shift depending on known examples of regulation [31]. Up to now, too few experimentally verified examples of TFs that regulate miRNAs are known, thus a model to introduce the "correct" time-shift could not be inferred. Furthermore, certain miRNAs were predicted to be clustered and share common promoter regions. Hence, a time-shift common to all miRNAs in a cluster was calculated for each of the associated TFs. As a criterion, common time-shifts were only taken into account if all *PCC*s between the TF and all miRNAs that form the cluster had the same sign (e.g. all positive or all negative) to avoid contradicting effects of the same TF on different miRNAs of the cluster. TF→miRNA associations where all considered time-shifts were discarded (because of sign disagreement) were excluded from further analysis.

### Identification of TFs central to regulation of miRNA genes

In order to find the TFs that have the most influence on miRNAs during the differentiation process, we analysed TFs corresponding to TF→miRNA associations having the highest absolute *PCC*. We ranked 1,989 TF→miRNA associations according to the absolute value of their corresponding *PCC*s. From the ranked associations we selected the upper quartile (with the highest absolute *PCCs*). In this manner, we obtained 498 associations with an absolute *PCC *greater than 0.775 (see Figure 3B). The 498 associations are formed by 111 unique TFs and 35 unique miRNAs. TFs that appear significantly more often in the upper quartile of associations are assumed to more likely play a central role in regulating miRNAs during the differentiation process. We utilised the one-sided Fisher's exact test to calculate the Bonferroni-corrected p-value for enrichment of each TF in the subset of 498 associations, in contrast to the remaining set of 1,491 associations. The correction factor is the number of unique TFs (258) in the complete set of all associations (1,989). In this manner, we found that 12 TFs are statistically significantly enriched in the set of 498 associations with a corrected p-value smaller than 0.01 (see Table 1). Six of these 12 TFs (ATF2, E2F3, HOXA4, NFE2L1, SP3, and YY1) have not been previously described as important for monocytic differentiation. The remaining TFs (namely, CEBPB [42], CREB1 [43], ELK1 [44], NFE2L2 [45], RUNX1 [42], and USF2 [46]) are known to play role in monocytic differentiation, but not explicitly as regulators of miRNAs in this process.

**Table 1:** TFs that are predicted to have a central role in regulating miRNAs

Gene Symbol	Gene ID	Hits in subset	Number of associations in subset	Total number of hits	Total number of associations	p-Value	p-Value (Bonferroni corrected)
CREB1	1385	18		20		1.33E-09	3.43E-07
ATF2	1386	15		17		6.56E-08	1.69E-05
SP3	6670	13		14		1.46E-07	3.76E-05
NFE2L2	4780	12		13		5.52E-07	1.42E-04
NFE2L1	4779	10		10		9.04E-07	2.33E-04
YY1	7528	10	498	11	1,989	7.72E-06	1.99E-03
CEBPB	1051	10		11		7.72E-06	1.99E-03
RUNX1	861	11		13		1.04E-05	2.69E-03
USF2	7392	9		10		2.85E-05	7.35E-03
E2F3	1871	13		18		3.18E-05	8.21E-03
ELK1	2002	10		12		3.59E-05	9.27E-03
HOXA4	3201	11		14		3.77E-05	9.74E-03

Table 1 shows the TFs that have been identified through statistical analysis to be statistically enriched (corrected p-value < 0.01) within the upper quartile of predicted TF→miRNA associations (see text). The correction factor utilised for the Bonferroni-correction is the number of unique TFs in the complete set of predicted TF→miRNA associations (258).

Our approach attempts to identify the most dominant TFs that putatively regulate miRNAs from the selected subset of TF→miRNA associations with highest *PCCs*. The complete set of 1,989 TF→miRNA associations consists of many associations with a low *PCC *(see Figure 3). In order to be able to focus on associations that are most likely genuine, we sub-selected the associations with the highest *PCCs*. At the same time we did not want to restrict the analysis to too few associations, so as to be able to deduce the general participants in the transcriptional regulation process of miRNAs. Consequently, we selected the upper quartile of TF→miRNA associations ranked based on decreasing absolute values of *PCC *as a reasonable compromise between sensitivity and specificity.

### Transcriptional circuitry of miRNAs during monocytic differentiation

**Figure 4:**
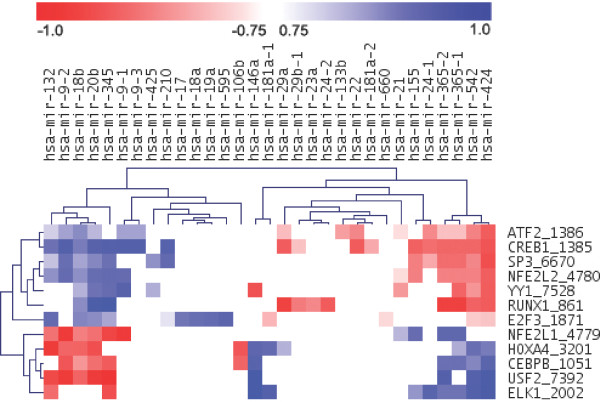
**Overview of 12 TFs and their regulatory effect on miRNA**. The figure presents a heat-map, with miRNA on the x-axis and TFs on the y-axis. The TF names on the y-axis are composed of the Entrez Gene symbol and Entrez Gene identifier, separated by "_". A coloured dot indicates the value of the *PCC *in expression between a TF and a mature miRNA where the TF has been predicted to regulate the corresponding miRNA. The figure only shows associations from the top quartile of associations with highest *PCC*. A white dot in the figure does not necessarily indicate a non-association. A possible association would have a *PCC *that prevented its inclusion in the top associations and is thus not shown. Furthermore, only TFs are shown that have been identified to play a central role in regulating miRNAs in the differentiation process. The heat-map has been clustered using hierarchical clustering with average linkage and Euclidian distance as the distance measure.

To shed light on a portion of the molecular underpinnings of monocytic differentiation we will discuss the TF→miRNA associations for miRNAs that have been described earlier to be affected by PMA stimulation. In this manner, we can confer whether or not our findings correspond to the published scientific findings and further introduce novel TF→miRNA associations. An overview of the regulatory effects of the TF subset (defined above) on the miRNAs is presented in Figure 4. The figure shows each association, from within the subset of the upper quartile of associations, in form of a coloured dot in a heat-map style of format using the TIGR Multiexperiment Viewer (version 4.3) (TMEV, [47]). We can observe certain clusters of miRNAs that are regulated by the same set of TFs. In the following discussion, we mainly focused on the upper quartile of TF→miRNA associations and on the TFs illustrated in Figure 4 that we have identified to be central to monocytic differentiation. For the sake of completeness, we also discuss several TFs that are known to be regulators of certain miRNAs, even though they might not appear in our set of "best" TF→miRNA associations. Subsets of miRNAs that have support through literature establishing their expression during PMA-induced differentiation are discussed. All network graphics in the following figures have been produced with the help of Cytoscape [48] and all pathway analyses were based on KEGG [49] using DAVID [50].

### miR-21

**Figure 5:**
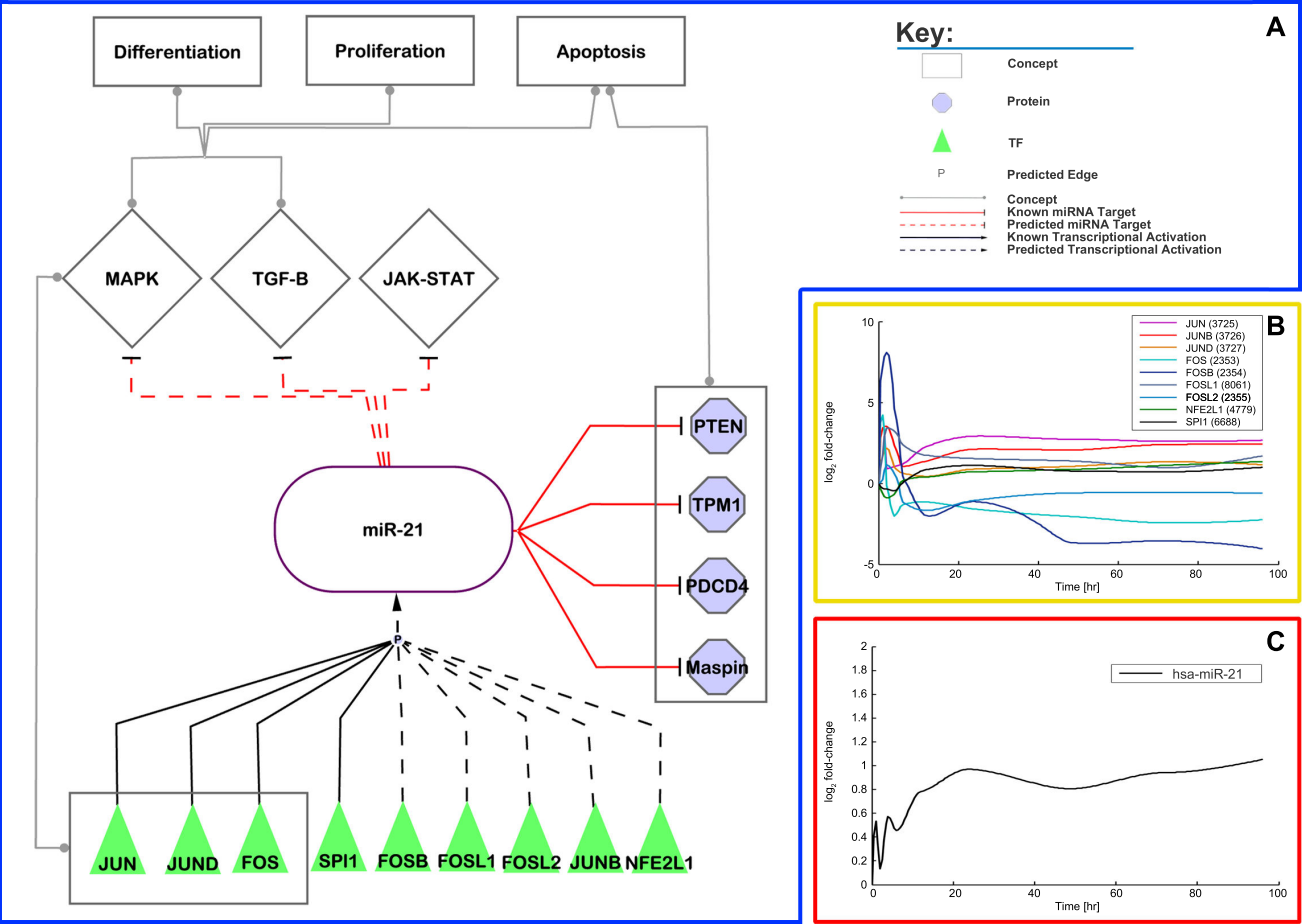
**Involvement of miR-21 in monocytic differentiation**. A/ Depicted are the predicted regulations of miR-21 and its involvement in the monocytic differentiation. B/ Depicted is the log_2 _*fc *over time of the interpolated expression data of selected TFs that are predicted to regulate miR-21. C/ Depicted is the log_2 _*fc *over time of the interpolated expression data of miR-21.

Fugita *et al*. demonstrated that mir-21 is expressed during PMA-induced differentiation in the human promyelocytic leukaemia cell line, HL-60 [51]. Our expression data demonstrate that miR-21 is up-regulated during the differentiation process (see Figure 5C). Our correlation data suggest that several of the 12 TFs (see above), which we identified as being central to the considered differentiation process bind in the promoter region of miR-21 (YY1, NFE2L2, ATF2 and NFE2L1, see Figure 4). Additionally, the binding of TFs, AP-1/c-jun, and c-fos to the promoter region of mir-21 has been demonstrated via chromatin immunoprecipitation (ChIP) in the human promyelocytic leukaemia cell line, HL-60 after 4 h PMA induction [51]. Our TFBS analysis results suggests the binding of several members of the JUN-FOS family (JUN, JUNB, JUND, FOS, FOSB, FOSL1, and FOSL2) to the promoter region of mir-21, even though they do not appear in the upper quartile of TF→miRNA associations. The expression data for the JUN family members displayed continued up-regulation for 96 hours, whereas FOS family members, with exception of FOSL1, were down-regulated after 4 hours (see Figure 5B). AP-1/c-jun form a complex with the JUN-FOS family members during transcription, and AP-1/c-jun is known to be activated by PMA induction which is supported by our findings (data not shown) [52]. Fugita *et al*. also demonstrated that AP-1 and SPI1 synergistically mediate the transcriptional process [51]. Our method predicted a SPI1 binding site in the promoter region of the mir-21 gene. The time-lagged expression correlation analysis demonstrated that SPI1 is highly correlated to miR-21 (*PCC *= 0.798; see Figures 5B and 5C).

miR-21 has been found to display anti-apoptotic functioning and targets tumour suppressor genes, like the PTEN gene in human hepatocellular cancer cells [53] and the tropomyosin 1 (TPM1), PDCD4, and maspin gene in the human breast cancer cell line, MDA-MB-231 [54]. The miR-21's predicted targets (see Methods) were found to be primarily involved in pathways such as TGF-β signalling pathway, MAPK signalling pathway and the JAK-STAT signalling pathway (see Figure 5A). The TGF-β signalling pathway and MAPK signalling pathway is primarily involved in differentiation, proliferation, apoptosis and developmental processes, while the JAK-STAT signalling pathway is involved in immune responses. We found that several TFs such as ATF2, FOS, JUN and JUND included in the predicted TF→mir-21 associations are involved in the MAPK signalling pathway (see Figure 5A).

Time-lagged expression correlation analysis demonstrated that NFE2L1 and SPI1 are highly correlated to miR-21 as opposed to YY1, NFE2L2, and ATF2, which have negative *PCC*s (see Figure 4). Besides JUN-FOS family members and SPI1 that are known to regulate the miR-21, our results suggest a novel NFE2L1→miR-21 association, which seems to play an important role in monocytic differentiation (see Figure 5A).

### miR-424

**Figure 6:**
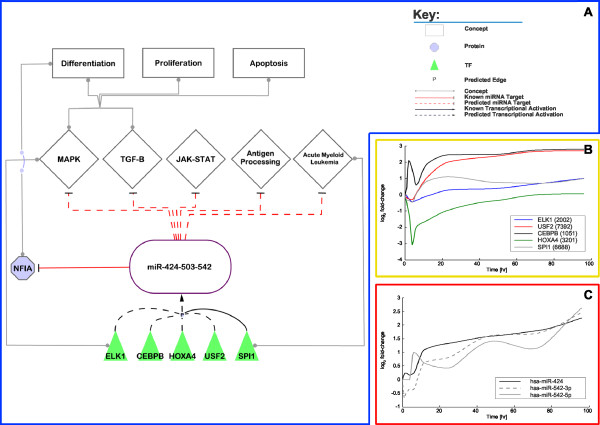
**Involvement of miR-424 in monocytic differentiation**. A/ Depicted are the predicted regulations of miR-424/miR-542/miR-503 and their involvement in the monocytic differentiation. B/ Depicted is the log_2 _*fc *over time of the interpolated expression data of selected TFs that are predicted to regulate miR-424/miR-542. C/ Depicted is the log_2 _*fc *over time of the interpolated expression data of miR-424, miR-542-3p, and miR-542-5p.

Rosa *et al*. reported that mir-424 is expressed during PMA-induced differentiation and that mir-424 is transcribed by SPI1 in the CD34+ human cord blood cells and CEBPA (C/EBPα) blocks SPI1 induced dendritic cell development from CD34^+ ^human cord blood cells by displacing the co-activator c-Jun [55,56]. The up-regulation of miR-424 (see Figure 6C) leads to the repression of NFIA which allows for the activation of differentiation specific genes such as M-CSFr (CSF1R) [55]. Furthermore, the pre-mir-424 is transcribed together with pre-mir-503 and pre-mir-542 as one transcript. These pre-miRNAs form the mature miRNAs miR-424, miR-503, miR-542-5p, and miR-542-3p. Our data suggest that several of the 12 TFs (see above), which we identified as being central to the considered differentiation process bind in the promoter region of miR-424 (RUNX1, E2F3, SP3, YY1, NFE2L2, CREB1, ATF2, USF2, ELK1, CEBPB and HOXA4; see Figure 4). Figure 4 shows that mir-424 and mir-542 are regulated by the same TFs and are thus as well clustered in the heat-map. However, mir-503, part of the same cluster and thus subject to the same regulations, is not displayed in Figure 4. This is a consequence of the expression data obtained for miR-503 causing the *PCC*s for the TF→miRNA associations to decrease and thus not being part of the top quartile of associations (see above). We further predicted a SPI1 and CEBPA binding site in the promoter region of these clustered miRNAs, which corresponds to findings reported by Rosa *et al*. [55]. SPI1 is positively correlated to miR-424 and CEBPA negatively. Furthermore, both associations are not within the top quartile of associations with highest *PCCs*. Nevertheless, these observations indicate that SPI1 enhances the expression of the mir-424 cluster and might work in conjunction with the other identified TFs to influence the miRNA's transcription.

The predicted targets of miR-424 were found to be involved in the same pathways as the targets of miR-21; the TGF-β signalling pathway, MAPK signalling pathway and JAK-STAT signalling pathway with additional pathways such as acute myeloid leukaemia and antigen processing and presentation, the p53 signalling pathway and SNARE interactions in vesicular transport. We found that several TFs included in the predicted TF→mir-424 associations, are involved in the MAPK signalling pathway (ELK1, ATF2), acute myeloid leukaemia (E2F3, RUNX1) and antigen processing and presentation (CREB1) (see Figure 6A).

The time-lagged expression correlation analysis demonstrated that of the 12 TFs (see above) only ELK1, USF2, CEBPB and HOXA4 were positively correlated to the expression of miR-424 (see Figure 4 and Figures 6B and 6C). Besides the earlier mentioned involvement of SPI1 in regulating mir-424 [55], our analysis suggests that ELK1, USF2, CEBPB and HOXA4 may be the TFs most likely responsible for the expression of mir-424 in monocytic differentiation (see Figure 6A).

### miR-155

**Figure 7:**
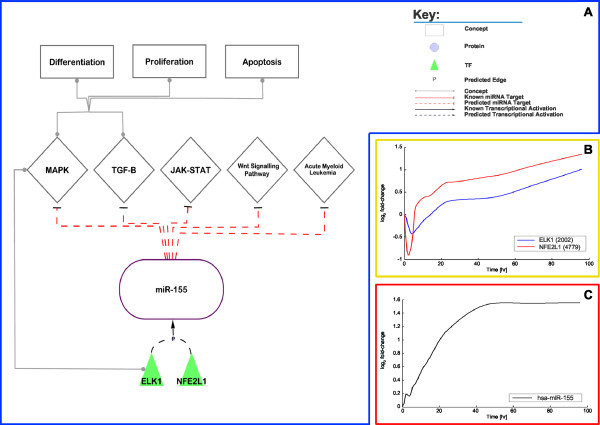
**Involvement of miR-155 in monocytic differentiation**. A/ Depicted are the predicted regulations of miR-155 and its involvement in the monocytic differentiation. B/ Depicted is the log_2 _*fc *over time of the interpolated expression data of selected TFs that are predicted to regulate miR-155. C/ Depicted is the log_2 _*fc *over time of the interpolated expression data of miR-155.

Chen *et al*. reported that mir-155 is expressed during PMA-induced differentiation in the human promyelocytic leukaemia cell line, HL-60 [57]. Our expression data demonstrate that miR-155 is up-regulated during the differentiation process (see Figure 7C). Our TFBS analysis data suggest that several of the 12 TFs (see above), which we identified as being central to the considered differentiation process, bind in the promoter region of miR-155 (SP3, NFE2L2, CREB1, NFE2L1 and ELK1; see Figure 4). Zeller *et al*. demonstrated binding of MYC to the promoter region of mir-155 in the human burkitt lymphoma cell line (P493-6) [58]. Also, Yin *et al*. demonstrated binding of FOSB and JUNB to the promoter region of mir-155 using chromatin immunoprecipitation (ChIP) in the human B-cell line [59]. miR-155 has been linked to Epstein-Barr virus (EBV) related diseases that are associated with latency during which only a subset of viral genes are transcribed with a set of EBV-encoded microRNAs. One such EBV gene is LMP1 which is a known oncogene that induces miR-155 in DeFew cells [60]. Gatto *et al*. demonstrated the positive expression of miR-155 in DeFew cells induced with PMA and that the promoter region has two NF-κB (NFKB1) binding sites [60]. Once again, our results predict the binding of several members of the JUN-FOS family to the promoter region of mir-155 but neither MYC nor NF-κB, this may be a consequent of the extracted regulatory region for mir-155, being incomplete. The expression data demonstrated the up-regulation of JUN-FOS (see Figure 5B) family members and NF-κB but a down-regulation of MYC (data not shown). Our observations indicate that JUN-FOS family enhances the expression of the miR-155 even though the predicted associations are not within the upper quartile of associations with highest *PCCs*.

MiR-155s predicted targets were found to be involved in the same pathways as the targets of miR-21 and miR-424; the TGF-β signalling pathway, MAPK signalling pathway and JAK-STAT signalling pathway with additional pathways such as acute myeloid leukaemia and Wnt signalling pathway (see Figure 7A). We found that several TFs such as ATF2 and ELK1, included in the predicted TF→mir-155 associations, are involved in the MAPK signalling pathway and CREB1 was found to be involved in antigen processing and presentation (see Figure 7A).

The time-lagged expression correlation analysis demonstrated that of the 12 TFs (see above) only NFE2L1 and ELK1 had TFBSs predicted within the promoter of miR-155 and were positively correlated to miR-155 (see Figure 4 and Figure 7B) and thus our findings propose that the NFE2L1→mir-155 and the ELK1→mir-155 associations are likely to be important to the monocytic differentiation process.

### miR-17-92

Members of the miRNA cluster mir-17-92 are known to be down regulated in the HL-60 cell line after PMA stimulation [57]. The miRNA cluster on chromosome 13 contains several miRNAs (hsa-mir-17, hsa-mir-18a, hsa-mir-19a, hsa-mir-20a, hsa-mir-19b-1, and hsa-mir-92-1 (hsa-mir-92-1 excluded from analysis, due to ambiguous nomenclature)) that are transcribed as a single transcript. Our data shows that members of miR-17-92 are indeed down regulated after PMA stimulation and furthermore, that the lowest *PCC *between the expression series of the miRNA cluster members is ~0.86, which supports the cluster membership. Even though the function of miR-17-92 is largely unknown, lymphomas that express these miRNAs at a high level have reduced apoptosis [61,62] and the miRNAs target multiple cell cycle regulators and promote G1→S phase transition [63]. Expression of miR-17-92 is high in proliferating cells and is positively regulated, in part, by MYC (c-Myc) [64]. E2F1, an activator of MYC, is itself a target of miR-17 and miR-20a [61] indicating that both MYC and E2F1 are under the control of a feedback loop. It has been experimentally shown that E2F3 activates the transcription of the miR-17-92 cluster [62,36]. A model has been proposed that miR-17-92 promotes cell proliferation by targeting pro-apoptotic E2F1 and thereby favouring proliferation through E2F3 mediated pathways [36]. Additionally, E2F3 is shown to be a predominant isoform that regulates miR-17-92 transcription [36]. We show that after ranking *PCC*s of gene expression between miRNAs and putative TFs, E2F3 is the only TF appearing significantly associated with miR-17-92 within the upper quartile of TF→miRNA associations (see Figure 4).

Amongst a small set of eight predicted regulators (E2F1, E2F3, E2F4, TFAP2A, TFAP2B, TFAP2C, TFDP1, SP1), TFDP1 is known to form a heterodimer with another putative TF, E2F1 [65], implicating TFDP1/E2F1 complex as a regulator of miR-17-92 transcription.

**Figure 8:**
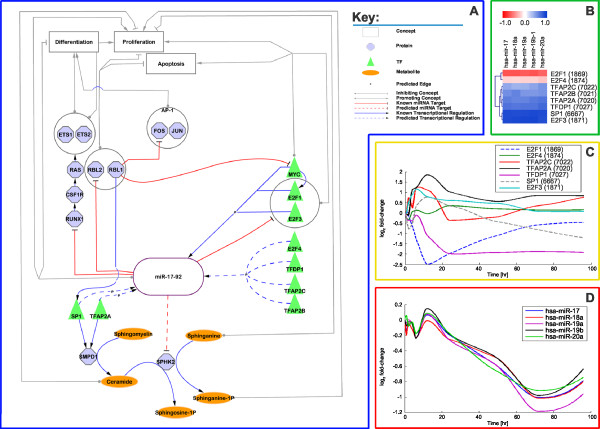
**Involvement of miR-17-92 in monocytic differentiation**. A/ Depicted are the predicted regulations of miR-17-92 and their involvement in the monocytic differentiation. B/ Depicted is a heat-map representation of the TFs that are predicted to regulate the miR-17-92 cluster. A coloured dot indicates the value of the *PCC *in expression between a TF and a miRNA where the TF has been predicted to regulate the miRNA. C/ Depicted is the log_2 _*fc *over time of the interpolated expression data of selected TFs that are predicted to regulate miR-17-92. D/ Depicted is the log_2 _*fc *over time of the interpolated expression data of miR-17-20a.

In Figure 8A we show the putative regulation of miR-17-92 and its known effects in proliferation, differentiation and apoptotic pathways. Specifically, we predict E2F1 and E2F3 to regulate the miR-17-92 cluster. Figure 8B shows that expression of miR-17-92 members are correlated to E2F3 with a minimum PCC of ~0.9. Conversely, miR-17-92 members are correlated with E2F1 by a maximum *PCC *of ~-0.65. A disproportionately high *PCC *of E2F3 gene expression to miR-17-92 as compared to other TFs seems to support the claims made by Woods *et. al*. that E2F3 is indeed the predominant TF in this regulatory context [36]. In addition, Cloonan *et al*. demonstrated that the pri-miRNA is cell cycle regulated, which supports the claim that the cluster is under the control of E2F family members, which are master regulators of the cell cycle [63]. On inspection of the log_2 _*fc *of TF gene expression over time (see Figure 8C) we observed that E2F3 is sharply up-regulated at 6 hours by ~2 fold, whilst its closely related and pro-apoptotic family member, E2F1, is down-regulated by a factor of ~5.7. After ~70 hours E2F3 and E2F1 gene expression levels return near to baseline, this corresponds to a progression towards a differentiated state before 96 hours post-PMA stimulation. Yet, regardless of the high *PCC *between E2F3 gene expression and the miR-17-92 cluster, the miRNA cluster is generally down-regulated (see Figure 8D). Acknowledging that the miRNA cluster targets and inhibits a well known RUNX1 (AML1) induced differentiation and proliferation pathway [66], these results strongly suggest that PMA stimulation disfavours both E2F1 induced proliferative and E2F1 induced apoptotic pathways. Whilst, equally, given that both ETS1 and ETS2, components of the above mentioned RUNX1 differentiation and proliferation pathway, are up-regulated (data not shown), these results indicate that PMA-treated monocytes up-regulate members of differentiation pathways. In light of the above findings we hypothesize, that since members of the AP-1 complex are concurrently up-regulated in the early stages after PMA stimulation, that monocytic differentiation is mediated by the M-CSF receptor-ligand RAS signalling pathway and indirectly controlled by miR-17-92 through the E2F TF family members E2F1 and E2F3. Generally, this hypothesis seems to be plausible, since RUNX1 is also an inhibitor of miR-17-92 [66] indicating its dual role to both suppress transcription of the pro-proliferative miRNA cluster miR-17-92, and to mediate an M-CSF receptor differentiation pathway. Additionally, patterns of expression observed for miR-17-92 during monocytic differentiation resemble a previous analysis of miR-17-92 expression levels during lung development [67] supporting the general involvement of miR-17-92 amongst differentiation pathways.

TFAP2A (AP-2) and SP1 are two TFs predicted to regulate the miR-17-92 cluster and are notably up-regulated along with the cluster in the first 20 hours post-PMA stimulation. TFAP2A and SP1 are known to activate transcription of an enzyme involved in the sphingolipid metabolism consisting of several metabolites known to affect cellular proliferation [68]. TFAP2A and SP1 transcribe sphingomyelin phosphodiesterase 1 (SMPD1) during monocytic differentiation in THP-1 cells after PMA stimuli [68]. SMPD1 is required for the cleavage of sphingomyelin to phosphocholine and ceramide. As ceramide is a known inhibitor of proliferation [69], it seems reasonable that TFs of SMPD1 are up-regulated during differentiation. However, ceramide is also a substrate for several other enzymes whose products have not been implicated in proliferation, apoptosis or differentiation. Interestingly, miR-19a and miR-19b (part of the miR-17-92 cluster), are predicted to target sphingosine kinase 2 (SPHK2) mRNA in four independent databases (see Methods). SPHK2 is an enzyme that metabolizes downstream ceramide products. In the sphingolipid metabolism, SPHK2 has two functions. First, it catalyses the production of sphingosine 1-phosphate from sphingosine, which is produced from ceramides; and second, it catalyses the production of sphinganine 1-phosphate from sphinganine [69]. Sphinganine and sphinganine 1-phosphate have been shown to inhibit and promote cell growth, respectively [69]. Thus, we note that the predicted targeting and down-regulation of SPHK2 by miR-19a and miR-19b in the first 20 hours post-PMA stimulation could prevent the metabolism of two anti-proliferative metabolites simultaneously, thereby inhibiting proliferation. It is known that PMA stimulation can block proliferation of THP-1 cells up to 24 hrs [4]. Thus, we propose an additional regulatory effect of TFAP2A and SP1 on the sphingolipid metabolism via the miRNA cluster miR-17-92. TFAP2A/SP1 mediated transcription of SMPD1 alone might not be enough to maintain an anti-proliferative ceramide signal, as ceramide is metabolized by other factors. On the other hand, TFAP2A/SP1 co-transcription of miRNAs targeting SPHK2 could provide an efficient and succinct means to retaining the ceramide signal.

### Summary

We have computationally analysed the regulatory machinery that potentially affects transcription of miRNA genes during monocytic differentiation. Our methodology included the extraction of promoter regions for miRNA genes defined by trimethylated histones, computational prediction of TFBSs to establish TF→miRNA associations, and the use of time-course expression data for TFs and miRNAs measured during monocytic differentiation to assess reliability of the predicted TF→miRNA associations via time-lagged expression correlation analysis.

Several TFs (CEBPB, CREB1, ELK1, NFE2L2, RUNX1, and USF2), which are known to play a role in monocytic differentiation, have been identified. Our analysis suggests that their role in the differentiation process could be further expanded through consideration of the transcriptional regulation of miRNAs they affect. In addition, we propose several TFs (NFE2L1, E2F3, ATF2, HOXA4, SP3, and YY1) to have a central role in the regulation of miRNA transcription during the differentiation process. We have shown for several miRNAs (miR-21, miR-155, miR-424, and miR-17-92) how their predicted transcriptional regulation could impact the differentiation process.

The process of identifying a complete list of TF→miRNA associations is hampered by the correct definition of promoter/regulatory regions being an unresolved issue that has a great impact on all studies that deal with gene regulation. We utilised a recent set of promoters defined based on the observation that histones are generally trimethylated at lysine 4 residues at TSSs of genes. Due to the employed definition of promoters by Marson *et al*. we find that for several miRNAs we were not able to extract regulatory regions. Furthermore, we note that the here utilised promoter regions defined by Marson *et al*. range in length between 200 and ~4,700 bp with 60 percent of the utilised promoter regions being of length below 202 bp. Consequently, the promoter set defined by Marson *et al*. allows us to mostly analyze regulatory elements proximal to the TSS. Nevertheless, it has been well documented [70,71] that proximal regulatory elements such as the TATA box play an important role in type II polymerase gene transcription. However, the utilised promoter set in this study represents one of the first sets of regulatory regions for miRNA genes.

It is important to note that the transcriptional circuitry described in our results is biased towards monocytic differentiation expression data, as several of TF→miRNA associations were discarded due to missing/incomplete expression data for either TF or miRNA. Furthermore, the expression based approach is limited in so far, as mature miRNAs are not the direct product of the TFs-mediated regulation but can undergo post-transcriptional regulation on pri- and pre-miRNA level [72]. Thus, it is possible that miRNAs that are transcribed together as one primary transcript, show different expression profiles on the mature miRNA level. The three main reasons that constrained the set of TF→miRNA associations we determined in this study are as follows: 1/ An incomplete promoter set for miRNA genes. 2/ An incomplete/inaccurate motif set for the prediction of TFBSs. 3/ An incomplete expression set for TFs and miRNAs. Each of the reasons impacts on the accuracy of the predicted TF→miRNA associations.

Nevertheless, our analysis provides the first large-scale insights into the transcriptional circuitry of miRNA genes in monocytic differentiation. Taken together, our results suggest important regulatory functions of several TFs on the transcriptional regulation of miRNAs. The regulatory networks discussed here form only the starting point for an in-depth analysis of the regulatory mechanisms involved. The predicted TF→miRNA associations and their corresponding *PCC*s can provide the basis for a more detailed experimental analysis of miRNA regulation during monocytic differentiation.

## Conclusions

We have computationally analysed the regulatory machinery that potentially controls the transcription of miRNA genes during monocytic differentiation. We made use of TFBS predictions in promoter regions of miRNA genes to associate TFs to miRNAs that they potentially regulate. With the help of time-course expression data for miRNAs and TFs during monocytic differentiation we evaluated each predicted association using a time-lagged expression correlation analysis. In this manner we derived a putative picture of the transcriptional circuitry that regulates miRNAs involved in human monocytic differentiation and determined potential key transcriptional regulators of miRNAs for this differentiation process.

## Methods

### miRNA time-course expression data

The miRNA expression profiles were obtained using Agilent's Human miRNA microarrays as described in [73]. Three biological replicates have been measured before PMA stimulation and post-PMA stimulation at nine time points ranging from 1-96 hrs (1 hr, 2 hr, 4 hr, 6 hr, 12 hr, 24 hr, 48 hr, 72 hr, 96 hr). We required that two criteria were met for the inclusion of a miRNA expression time-series in the analysis:

i/ Expression of each miRNA should be denoted as "present" in at least one time point. Otherwise we assume that the expression series for the miRNA is insignificant.

ii/ For a miRNA, i/ must hold true in at least two of the three biological replicates.

The expression values of different biological replicates for a miRNA that satisfy the criteria have been averaged at each time point to generate one expression series per miRNA. Finally, each expression series was interpolated using piecewise cubic hermite interpolation [74,75] with half an hour steps. In this manner, we obtained 193 (0-96 hrs) expression values for each individual miRNA expression series.

### Identification of miRNAs showing differential gene expression

We calculate the log_2 _*fc *by dividing each expression value of a miRNA by its expression value at zero hour (control) and taking the logarithm of base two (log_2_) of that ratio. A miRNA is considered to be influenced by the PMA stimulation in the differentiation process, if

i/ In at least one time point *t *its log_2 _*fc *> 1 or log_2 _*fc *< -1.

ii/ At any time point *t *where i/ holds true, the absolute difference *d*_t _in expression *e*_t _at time point *t *and the expression *e*_0 _at zero hours must be greater than 0.1.

### Transcription factor time-course expression data

The TF expression profiles were obtained using qRT-PCR as described in [6,76]. Two biological replicates have been measured prior to PMA stimulation and in nine time points post-PMA simulation (1 hr, 2 hr, 4 hr, 6 hr, 12 hr, 24 hr, 48 hr, 72 hr, 96 hr). Primer design, RNA preparation, and cDNA synthesis have been performed analogously to [76]. Normalization of the expression data of both replicates have been done as described in [6,77]. All expression series for a TF that had available expression data within two biological replicates have been averaged over the respective biological replicates to produce one series of expression values per TF. Finally, each expression series was interpolated in half an hour steps using piecewise cubic hermite interpolation. Thus, we obtain 193 (0-96 hrs) expression values for each individual TF expression series.

### Defining the promoter regions for miRNAs

We adopted the definition of miRNA promoters from [30]. Each of the promoter regions had a score associated (as defined in [30]) that represents the confidence of dealing with a genuine regulatory region. We extracted all promoter regions with a score greater or equal to zero. The coordinates of the promoter regions were translated from the Human genome build 17 (hg17) to the Human genome build 18 (hg18) [78] using the UCSC liftover program [79].

### TFBS analysis of miRNA promoter regions

TFBSs were mapped to the promoter region of the miRNAs with the MATCH™ program [80] using 522 mammalian matrices of TRANSFAC Professional Database (version 11.4) with their corresponding minimum false positive threshold profiles. Since TRANSFAC matrices are frequently associated with several TFs whose binding sites were used in building these matrices, we associated to each matrix all respective TFs (that have an Entrez Gene identifier associated). For example, we can associate several members of the JUN-FOS family (JUN, JUNB, JUND, FOS, FOSB, etc.) to matrix M00517. Binding sites of these TFs have been utilised to create this matrix. Thus, all of the TFs might be able to bind the TFBS predicted by the matrix.

### Weighting associations using Pearson correlation

For each of the predicted TF→miRNA associations, scores (*PCCs*) were calculated as an indicator of how reliable the predicted association is, and as a measure of the strength of the association within the context of monocytic differentiation. The expression data for TFs and mature miRNAs during monocytic differentiation were utilised to calculate the best time-lagged expression correlation for a TF→miRNA association. The time-lagged expression correlation analysis calculates *PCC *between the TF expression and the time-shifted mature miRNA expression at different time-delays in order to take the influence of the TF on the miRNA transcription over time into account. We find the time-delay that maximizes the absolute value of *PCC *between the expression of the TF and that of the mature miRNA. The associations between pre-miRNA and the mature miRNA have been extracted using miRBase sequence database (version 10.1) [81,13,14].

For each predicted TF→miRNA association, where the miRNA does not share the same promoter with other miRNAs (i.e. not in a cluster), we calculate the *PCC *as follows:

i/ Identify the time-shift *s*_t_. This is the time-shift where the absolute value of the *PCC *between the expression of the TF and the respective mature miRNA is maximal. We calculate the *PCC *for time-shifts ranging from 0.5 hour to six hours in intervals of half an hour.

ii/ The *PCC *for the association is calculated as *PCC *of the expression of TF and mature miRNA at the time-shift *s*_t_found in i/.

If a miRNA appears in a cluster with other miRNAs on the genome, then the predicted TF in the promoter of that cluster is associated to each of the respective miRNAs. Since the cluster is transcribed as one primary transcript we assume that a TF regulates each miRNA within the cluster with the same time-shift. Thus, we calculate one common time-shift *s*_t _for the considered TF and all miRNAs within the cluster. The time-shift *s*_t _is calculated as follows:

i/ The *PCC *of expression between the TF and each miRNA in the cluster is calculated for each considered time-shift (0.5 hour to six hours).

ii/ The average of all *PCC*s derived in i/ was calculated for each time-shift (0.5 hour to six hours). As a criterion for inclusion, the calculated *PCC*s for all associations should to have the same sign.

iii/ If ii/ could not be calculated at any time-shift (due to the sign rule), we did not assume that the TF *X *regulates any miRNA in that cluster and all *X*→miRNA associations of that cluster were discarded.

iv/ If not iii/, then the time-shift *s*_t _is determined as the time-shift that maximizes the average calculated in ii/.

*PCC *of one TF→miRNA association where the miRNA is part of a cluster forms the *PCC *of expression of the TF and the respective mature miRNA at the determined time-shift *s*_t _for the TF and the cluster. If a pre-miRNA is associated to more than one mature miRNA from its 5' and 3' arm, then the *PCC *is calculated independently for each mature miRNA and the maximum *PCC *is chosen.

### Target predictions of miRNAs

The target gene predictions of human miRNAs have been gathered from four public available databases for miRNA target predictions (microRNA.org version 4 [15], TargetScan version 4.2 [9], miRBase version 5 [13,14], and EIMMO2 [16] with a cut-off value greater than 0.5). All target gene identifiers utilised in the respective databases have been converted to Entrez Gene identifiers using BioMart [82]. If this was not possible the prediction has been discarded. We considered only predictions that are present in at least three out of the four databases.

## Abbreviations

TF: transcription factor; TFBS: transcription factor binding site; TSS: transcription start site; PCC: Pearson correlation coefficient; fc: fold-change;

## Authors' contributions

SS and VBB conceptualized the study and wrote the paper; SS performed the analysis; CRM, ME, MK, and US performed parts of the analysis; HS and YH contributed experimental data. All authors have read and approved the final version of the manuscript.

## Supplementary Material

Additional file 1 **Promoter regions miRNAs**. The first column contains the pre-miRNA identifier. The second column contains the chromosomal positions of the associated promoter regions separated by comma. Note that several pre-miRNAs can have the same promoter region associated, because they are transcribed together in form of a cluster. Click here for file

Additional file 2 **Interpolated expression data for 34 mature miRNA**. The file consists of 195 columns. The first column contains the mature miRNA identifier. The second column contains the associated pre-miRNA identifier(s). Column 3-194 contain the interpolated expression values ranging in half an hour steps from 0 to 96 hours. Click here for file

Additional file 3 **Predicted TF→miRNA associations and their inferred *PCC *values**. The file consists of three columns. The first column contains the TF. An identifier consists of the Gene Symbol separated by an underscore with the Entrez Gene id. The second column contains the pre-miRNA identifier of the miRNA that forms an association with the TF of the first column. The third column contains the inferred *PCC *for the association, which is based on the expression data of the TF and the mature miRNA associated to the pre-miRNA(s). In total the file contains 1,989 TF→miRNA associations. Click here for file

Additional file 4 **Interpolated expression data for 258 TFs**. The file consists of interpolated expression data for 258 TFs that are present in the predicted TF→miRNA associations. Furthermore, the file consists of 194 columns. The first column is the TF identifier (Entrez Gene Id). Column 2-194 contain the interpolated expression values ranging in half an hour steps from 0 to 96 hours. Click here for file

## References

[B1] van Furth R Cohn ZA Hirsch JG The mononuclear phagocyte system: a new classification of macrophages, monocytes, and their precursor cells Bull World Health Organ 1972 10 845 52 4538544 PMC2480884

[B2] Tsuchiya S Yamabe M Yamaguchi Y Establishment and characterization of a human acute monocytic leukemia cell line (THP-1) Int J Cancer 1980 10 171 6 10.1002/ijc.2910260208 6970727

[B3] Auwerx J The human leukemia cell line, THP-1: a multifacetted model for the study of monocyte-macrophage differentiation Experientia 1991 10 22 31 10.1007/BF02041244 1999239

[B4] Traore K Trush MA George M Signal transduction of phorbol 12-myristate 13-acetate (PMA)-induced growth inhibition of human monocytic leukemia THP-1 cells is reactive oxygen dependent Leuk Res 2005 10 863 79 10.1016/j.leukres.2004.12.011 15978937

[B5] Martinez FO Gordon S Locati M Mantovani A Transcriptional profiling of the human monocyte-to-macrophage differentiation and polarization: new molecules and patterns of gene expression J Immunol 2006 10 7303 11 17082649 10.4049/jimmunol.177.10.7303

[B6] Suzuki H Forrest ARR van Nimwegen E The transcriptional network that controls growth arrest and differentiation in a human myeloid leukemia cell line Nat Genet 2009 10 5 553 62 10.1038/ng.375 19377474 PMC6711855

[B7] Lee C Risom T Strauss WM MicroRNAs in mammalian development Birth Defects Res C Embryo Today 2006 10 129 39 10.1002/bdrc.20072 16847889

[B8] Bartel DP MicroRNAs: genomics, biogenesis, mechanism, and function Cell 2004 10 281 97 10.1016/S0092-8674(04)00045-5 14744438

[B9] Lewis BP Shih I Jones-Rhoades MW Bartel DP Burge CB Prediction of mammalian microRNA targets Cell 2003 10 787 98 10.1016/S0092-8674(03)01018-3 14697198

[B10] Grimson A Farh KK Johnston WK MicroRNA Targeting Specificity in Mammals: Determinants beyond Seed Pairing Molecular Cell 2007 10 91 105 10.1016/j.molcel.2007.06.017 17612493 PMC3800283

[B11] Lewis BP Burge CB Bartel DP Conserved seed pairing, often flanked by adenosines, indicates that thousands of human genes are microRNA targets Cell 2005 10 15 20 10.1016/j.cell.2004.12.035 15652477

[B12] John B Enright AJ Aravin A Human MicroRNA Targets PLoS Biology 2004 10 e363 10.1371/journal.pbio.0020363 15502875 PMC521178

[B13] Griffiths-Jones S Grocock RJ van Dongen S Bateman A Enright AJ miRBase: microRNA sequences, targets and gene nomenclature Nucl Acids Res 2006 10 D140 144 10.1093/nar/gkj112 16381832 PMC1347474

[B14] Griffiths-Jones S Saini HK van Dongen S Enright AJ miRBase: tools for microRNA genomics Nucl Acids Res 2008 10 D154 158 10.1093/nar/gkm952 17991681 PMC2238936

[B15] Betel D Wilson M Gabow A Marks DS Sander C The microRNA.org resource: targets and expression Nucl Acids Res 2008 10 D149 153 10.1093/nar/gkm995 18158296 PMC2238905

[B16] Gaidatzis D van Nimwegen E Hausser J Zavolan M Inference of miRNA targets using evolutionary conservation and pathway analysis BMC Bioinformatics 2007 10 69 10.1186/1471-2105-8-69 17331257 PMC1838429

[B17] Bracht J Hunter S Eachus R Weeks P Pasquinelli AE Trans-splicing and polyadenylation of let-7 microRNA primary transcripts RNA 2004 10 1586 94 10.1261/rna.7122604 15337850 PMC1370645

[B18] Cai X Hagedorn CH Cullen BR Human microRNAs are processed from capped, polyadenylated transcripts that can also function as mRNAs RNA 2004 10 1957 66 10.1261/rna.7135204 15525708 PMC1370684

[B19] Lee Y Kim M Han J MicroRNA genes are transcribed by RNA polymerase II EMBO J 2004 10 4051 60 10.1038/sj.emboj.7600385 15372072 PMC524334

[B20] Denli AM Tops BBJ Plasterk RHA Ketting RF Hannon GJ Processing of primary microRNAs by the Microprocessor complex Nature 2004 10 231 5 10.1038/nature03049 15531879

[B21] Gregory RI Yan K Amuthan G The Microprocessor complex mediates the genesis of microRNAs Nature 2004 10 235 40 10.1038/nature03120 15531877

[B22] Bohnsack MT Czaplinski K Gorlich D Exportin 5 is a RanGTP-dependent dsRNA-binding protein that mediates nuclear export of pre-miRNAs RNA 2004 10 185 91 10.1261/rna.5167604 14730017 PMC1370530

[B23] Lee Y Ahn C Han J The nuclear RNase III Drosha initiates microRNA processing Nature 2003 10 415 9 10.1038/nature01957 14508493

[B24] Lee Y Jeon K Lee J Kim S Kim VN MicroRNA maturation: stepwise processing and subcellular localization EMBO J 2002 10 4663 70 10.1093/emboj/cdf476 12198168 PMC126204

[B25] Lau NC Lim LP Weinstein EG Bartel DP An abundant class of tiny RNAs with probable regulatory roles in Caenorhabditis elegans Science 2001 10 858 62 10.1126/science.1065062 11679671

[B26] Lagos-Quintana M Rauhut R Lendeckel W Tuschl T Identification of novel genes coding for small expressed RNAs Science 2001 10 853 8 10.1126/science.1064921 11679670

[B27] Xie Z Allen E Fahlgren N Expression of Arabidopsis MIRNA Genes Plant Physiol 2005 10 2145 2154 10.1104/pp.105.062943 16040653 PMC1183402

[B28] Guenther MG Levine SS Boyer LA Jaenisch R Young RA A chromatin landmark and transcription initiation at most promoters in human cells Cell 2007 10 77 88 10.1016/j.cell.2007.05.042 17632057 PMC3200295

[B29] Barski A Cuddapah S Cui K High-resolution profiling of histone methylations in the human genome Cell 2007 10 823 37 10.1016/j.cell.2007.05.009 17512414

[B30] Marson A Levine SS Cole MF Connecting microRNA genes to the core transcriptional regulatory circuitry of embryonic stem cells Cell 2008 10 521 33 10.1016/j.cell.2008.07.020 18692474 PMC2586071

[B31] Shi Y Mitchell T Bar-Joseph Z Inferring pairwise regulatory relationships from multiple time series datasets Bioinformatics 2007 10 755 763 10.1093/bioinformatics/btl676 17237067

[B32] Arkin A Shen P Ross J A Test Case of Correlation Metric Construction of a Reaction Pathway from Measurements Science 1997 10 1275 1279 10.1126/science.277.5330.1275

[B33] Redestig H Weicht D Selbig J Hannah M Transcription factor target prediction using multiple short expression time series from Arabidopsis thaliana BMC Bioinformatics 2007 10 454 10.1186/1471-2105-8-454 18021423 PMC2198923

[B34] Lee HK Hsu AK Sajdak J Qin J Pavlidis P Coexpression Analysis of Human Genes Across Many Microarray Data Sets Genome Res 2004 10 1085 1094 10.1101/gr.1910904 15173114 PMC419787

[B35] Schmitt WA Raab RM Stephanopoulos G Elucidation of Gene Interaction Networks Through Time-Lagged Correlation Analysis of Transcriptional Data Genome Res 2004 10 1654 1663 10.1101/gr.2439804 15289483 PMC509275

[B36] Woods K Thomson JM Hammond SM Direct regulation of an oncogenic micro-RNA cluster by E2F transcription factors J Biol Chem 2007 10 2130 4 10.1074/jbc.C600252200 17135268

[B37] Wingender E Chen X Fricke E The TRANSFAC system on gene expression regulation Nucleic Acids Res 2001 10 281 3 10.1093/nar/29.1.281 11125113 PMC29801

[B38] Matys V Kel-Margoulis OV Fricke E TRANSFAC and its module TRANSCompel: transcriptional gene regulation in eukaryotes Nucleic Acids Res 2006 10 D108 10 10.1093/nar/gkj143 16381825 PMC1347505

[B39] Wu W Li W Chen B Identifying regulatory targets of cell cycle transcription factors using gene expression and ChIP-chip data BMC Bioinformatics 2007 10 188 10.1186/1471-2105-8-188 17559637 PMC1906835

[B40] Yu H Luscombe NM Qian J Gerstein M Genomic analysis of gene expression relationships in transcriptional regulatory networks Trends Genet 2003 10 422 7 10.1016/S0168-9525(03)00175-6 12902159

[B41] Qian J Dolled-Filhart M Lin J Yu H Gerstein M Beyond synexpression relationships: local clustering of time-shifted and inverted gene expression profiles identifies new, biologically relevant interactions J Mol Biol 2001 10 1053 66 10.1006/jmbi.2000.5219 11743722

[B42] Valledor AF Borràs FE Cullell-Young M Celada A Transcription factors that regulate monocyte/macrophage differentiation J Leukoc Biol 1998 10 405 17 9544570 10.1002/jlb.63.4.405

[B43] Sawka-Verhelle D Escoubet-Lozach L Fong AL PE-1/METS, an antiproliferative Ets repressor factor, is induced by CREB-1/CREM-1 during macrophage differentiation J Biol Chem 2004 10 17772 84 10.1074/jbc.M311991200 14754893

[B44] Li C Yu Y Wang Y Both ERK and JNK are required for enhancement of MD-2 gene expression during differentiation of HL-60 cells Biol Cell 2008 10 365 75 10.1042/BC20070140 18181766

[B45] Gavin IM Glesne D Zhao Y Kubera C Huberman E Spermine acts as a negative regulator of macrophage differentiation in human myeloid leukemia cells Cancer Res 2004 10 7432 8 10.1158/0008-5472.CAN-04-0051 15492267

[B46] Chen N Szentirmay MN Pawar SA Tumor-suppression function of transcription factor USF2 in prostate carcinogenesis Oncogene 2006 10 579 87 16186802 10.1038/sj.onc.1209079

[B47] Saeed AI Bhagabati NK Braisted JC TM4 microarray software suite Methods Enzymol 2006 10 134 93 10.1016/S0076-6879(06)11009-5 16939790

[B48] Shannon P Markiel A Ozier O Cytoscape: a software environment for integrated models of biomolecular interaction networks Genome Res 2003 10 2498 504 10.1101/gr.1239303 14597658 PMC403769

[B49] Kanehisa M Araki M Goto S KEGG for linking genomes to life and the environment Nucleic Acids Res 2008 10 D480 4 10.1093/nar/gkm882 18077471 PMC2238879

[B50] Dennis G Sherman BT Hosack DA DAVID: Database for Annotation, Visualization, and Integrated Discovery Genome Biol 2003 10 P3 10.1186/gb-2003-4-5-p3 12734009 PMC193660

[B51] Fujita S Ito T Mizutani T miR-21 Gene expression triggered by AP-1 is sustained through a double-negative feedback mechanism J Mol Biol 2008 10 492 504 10.1016/j.jmb.2008.03.015 18384814

[B52] Mollinedo F Gajate C Tugores A Flores I Naranjo JR Differences in expression of transcription factor AP-1 in human promyelocytic HL-60 cells during differentiation towards macrophages versus granulocytes Biochem J 1993 10 Pt 1 137 44 8363564 10.1042/bj2940137 PMC1134576

[B53] Meng F Henson R Wehbe-Janek H MicroRNA-21 regulates expression of the PTEN tumor suppressor gene in human hepatocellular cancer Gastroenterology 2007 10 647 58 10.1053/j.gastro.2007.05.022 17681183 PMC4285346

[B54] Zhu S Wu H Wu F MicroRNA-21 targets tumor suppressor genes in invasion and metastasis Cell Res 2008 10 350 9 10.1038/cr.2008.24 18270520

[B55] Rosa A Ballarino M Sorrentino A The interplay between the master transcription factor PU.1 and miR-424 regulates human monocyte/macrophage differentiation Proc Natl Acad Sci USA 2007 10 19849 54 10.1073/pnas.0706963104 18056638 PMC2148386

[B56] Reddy VA Iwama A Iotzova G Granulocyte inducer C/EBPalpha inactivates the myeloid master regulator PU.1: possible role in lineage commitment decisions Blood 2002 10 483 90 10.1182/blood.V100.2.483 12091339

[B57] Chen A Luo M Yuan G Complementary analysis of microRNA and mRNA expression during phorbol 12-myristate 13-acetate (TPA)-induced differentiation of HL-60 cells Biotechnol Lett 2008 10 2045 52 10.1007/s10529-008-9800-8 18648749

[B58] Zeller KI Zhao X Lee CWH Global mapping of c-Myc binding sites and target gene networks in human B cells Proc Natl Acad Sci USA 2006 10 17834 9 10.1073/pnas.0604129103 17093053 PMC1635161

[B59] Yin Q Wang X McBride J Fewell C Flemington E B-cell receptor activation induces BIC/miR-155 expression through a conserved AP-1 element J Biol Chem 2008 10 2654 62 10.1074/jbc.M708218200 18048365 PMC2810639

[B60] Gatto G Rossi A Rossi D Epstein-Barr virus latent membrane protein 1 trans-activates miR-155 transcription through the NF-{kappa}B pathway Nucleic Acids Res 2008 10 6608 6619 10.1093/nar/gkn666 18940871 PMC2582607

[B61] He L Thomson JM Hemann MT A microRNA polycistron as a potential human oncogene Nature 2005 10 828 33 10.1038/nature03552 15944707 PMC4599349

[B62] Sylvestre Y De Guire V Querido E An E2F/miR-20a autoregulatory feedback loop J Biol Chem 2007 10 2135 43 10.1074/jbc.M608939200 17135249

[B63] Cloonan N Brown M Steptoe A The miR-17-5p microRNA is a key regulator of the G1/S phase cell cycle transition Genome Biol 2008 10 R127 10.1186/gb-2008-9-8-r127 18700987 PMC2575517

[B64] O'Donnell KA Wentzel EA Zeller KI Dang CV Mendell JT c-Myc-regulated microRNAs modulate E2F1 expression Nature 2005 10 839 43 10.1038/nature03677 15944709

[B65] Helin K Wu CL Fattaey AR Heterodimerization of the transcription factors E2F-1 and DP-1 leads to cooperative trans-activation Genes Dev 1993 10 1850 61 10.1101/gad.7.10.1850 8405995

[B66] Fontana L Pelosi E Greco P MicroRNAs 17-5p-20a-106a control monocytopoiesis through AML1 targeting and M-CSF receptor upregulation Nat Cell Biol 2007 10 775 87 10.1038/ncb1613 17589498

[B67] Lu Y Thomson JM Wong HYF Hammond SM Hogan BL Transgenic over-expression of the microRNA miR-17-92 cluster promotes proliferation and inhibits differentiation of lung epithelial progenitor cells Developmental Biology 2007 10 442 453 10.1016/j.ydbio.2007.08.007 17765889 PMC2052923

[B68] Langmann T Buechler C Ries S Transcription factors Sp1 and AP-2 mediate induction of acid sphingomyelinase during monocytic differentiation J Lipid Res 1999 10 870 880 10224156

[B69] Merrill AH De Novo Sphingolipid Biosynthesis: A Necessary, but Dangerous, Pathway J Biol Chem 2002 10 25843 25846 10.1074/jbc.R200009200 12011104

[B70] Sandelin A Carninci P Lenhard B Mammalian RNA polymerase II core promoters: insights from genome-wide studies Nat Rev Genet 2007 10 424 36 10.1038/nrg2026 17486122

[B71] Carninci P Sandelin A Lenhard B Genome-wide analysis of mammalian promoter architecture and evolution Nat Genet 2006 10 626 35 10.1038/ng1789 16645617

[B72] Obernosterer G Leuschner PJF Alenius M Martinez J Post-transcriptional regulation of microRNA expression RNA 2006 10 1161 7 10.1261/rna.2322506 16738409 PMC1484437

[B73] Forrest ARR Kanamori-Katayama M Tomaru Y Induction of microRNAs mir-155, mir-222, mir-424 and mir-503, promotes monocytic differentiation through combinatorial regulation Leukemia in press 10.1038/leu.2009.246 19956200

[B74] Fritsch FN Carlson RE Monotone Piecewise Cubic Interpolation SIAM J Numerical Analysis 1980 10 238 246 10.1137/0717021

[B75] Kahaner DK Moler C Nash SG Numerical Methods and Software 1988 Prentice-Hall

[B76] Suzuki H Okunishi R Hashizume W Identification of region-specific transcription factor genes in the adult mouse brain by medium-scale real-time RT-PCR FEBS Lett 2004 10 214 8 10.1016/j.febslet.2004.07.068 15328000

[B77] Mar JC Kimura Y Schroder K Data-driven normalization strategies for high-throughput quantitative RT-PCR BMC Bioinformatics 2009 10 110 10.1186/1471-2105-10-110 19374774 PMC2680405

[B78] Initial sequencing and analysis of the human genome Nature 2001 10 860 921 10.1038/35057062 11237011

[B79] Hinrichs AS Karolchik D Baertsch R The UCSC Genome Browser Database: update 2006 Nucl Acids Res 2006 10 D590 598 10.1093/nar/gkj144 16381938 PMC1347506

[B80] Kel A Gossling E Reuter I MATCHTM: a tool for searching transcription factor binding sites in DNA sequences Nucleic Acids Res 2003 10 3576 3579 10.1093/nar/gkg585 12824369 PMC169193

[B81] Griffiths-Jones S The microRNA Registry Nucleic Acids Res 2004 10 D109 11 10.1093/nar/gkh023 14681370 PMC308757

[B82] Durinck S Moreau Y Kasprzyk A BioMart and Bioconductor: a powerful link between biological databases and microarray data analysis Bioinformatics 2005 10 3439 40 10.1093/bioinformatics/bti525 16082012

